# Gender differences in quality of dying and death among older adults: a cross-sectional study in China

**DOI:** 10.3389/fpubh.2025.1542918

**Published:** 2025-03-05

**Authors:** Xiaohong Feng, Shumei Liang, Xiujun Dai, Jinlin Du, Zheng Yang

**Affiliations:** School of Public Health, Guangdong Medical University, Dongguan, China

**Keywords:** quality of dying and death, older adults, gender differences, Fairlie decomposition analysis, CLHLS

## Abstract

**Background:**

The aging of China is deepening year by year, and improving the quality of dying and death (QODD) is increasingly becoming an urgent and realistic need. This study explores the gender differences in the quality of dying and death and its influencing factors among Chinese older adults, aiming to provide assistance to the relevant authorities in formulating end-of-life care policies for the older adults, and to adapt to the needs of an aging society.

**Methods:**

Based on the data of the Chinese Longitudinal Health Longevity Survey (CLHLS) during 2008–2018, a total of 7,341 respondents were included. Chi-square test and logistic regression analysis were used to analyze the quality of dying and death among Chinese older adults and its influencing factors. In addition, A Fairlie decomposition analysis (FDA) was conducted to ascertain the degree of influence exerted by various contributing factors.

**Results:**

The proportion of high QODD among female older adults (63.80%) was significantly higher than male older adults (56.00%), which was statistically significant. Logistic regression showed that age, residence, home facilities score, place of death, medical costs, got timely treatment, number of chronic diseases and unconsciousness were the factors influencing QODD among male older adults. Meanwhile, residence, marital status, home facilities score, place of death, got timely treatment, bedridden, suffered from serious illness, unconsciousness and drinking were the factors influencing QODD among female older adults. FDA showed that 47.89% of the differences in QODD were caused by the observed variables, while 52.11% of the differences were caused by gender differences and unmeasured variables.

**Conclusion:**

Chinese men have a poorer QODD compared to women. The main factors contributing to this difference were age, the number of chronic diseases, suffered from serious illness, unconsciousness, place of death, residence and home facilities scores. To ensure successful aging, the relevant departments should focus on these factors and work toward reducing the gender differences in QODD.

## 1 Introduction

The global population is undergoing a demographic transition, with an increasing proportion of individuals belonging to the older age groups. According to data from the World Population Prospects 2022, the population aged 65 and over is growing at a faster rate than that of individuals under 65. By 2050, it is projected that the share of the global population aged 65 and over will increase from 10 to 16% (1). Simultaneously, the population aged 60 and over in China is also experiencing rapid growth, with estimates suggesting it will exceed 420 million by 2035, representing over 30% of the total population (2). The aging population and the increasing number of older individuals pose significant challenges to the sustainability of the social security system and the provision of public services.

In response to this challenge, the Chinese government has proposed to improve national health policies to ensure the provision of comprehensive health services throughout the life course. Among these initiatives, enhancing the quality of dying and death (QODD) is a significant objective (3). QODD refers to the degree to which a person’s preferences for dying and the moment of death are consistent with others’ observations of how the person actually died (4). It reflects the consistency between individuals’ end-of-life experiences and expectations (5). Research efforts have been directed toward understanding the current status of QODD and the factors that affect it. A cross-country study involving 81 nations revealed that the United Kingdom topped the rankings, while China was positioned 53rd (6). Findings from Liu et al. indicated that the QODD score for Chinese patients with end-stage cancer was (52.71 ± 17.51), which is relatively low, compared to the scores of the United States (67.1 ± 25.9) and Israel (57.2 ± 15.0) (7–9). Literature suggests that the QODD of terminally ill patients is influenced by a multitude of factors, including age, gender, religion, disease type, comorbidities, place of death, and family support (7, 10–13).

Gender disparities are a critical focus in health filed research (14–17). Some studies have shown that older men have a better quality of life than older women (18–20). On the other hand, it has been shown that women have a higher quality of life (21, 22). Furthermore, additional studies showed no difference in quality of life between male and female older adults (23, 24). Consistently, studies have shown that males and females exhibit different patterns of morbidity, life expectancy, and health behaviors, which may influence their end-of-life experiences and the quality of death (25). Secondly, women, who often outlive men, may spend more years with disabilities and chronic illnesses (26). This gender disparity in health outcomes raises questions about the quality of life and the quality of death for older women compared to their male counterparts. Moreover, gender roles and societal expectations can influence the provision and receipt of end-of-life care (27–29), potentially resulting in differing dying experiences between genders. Despite the recognition of these gender differences, there is a paucity of research specifically addressing how gender influences the quality of death among the older adults.

Therefore, this study aims to employ the Fairlie decomposition method to investigate the influence of gender disparities on the QODD of older adults and to ascertain the extent to which each factor contributes to this phenomenon, in an attempt to provide practical references for relevant authorities to formulate end-of-life care policies for the older adults in order to improve the QODD of the older adults, as well as to promote the development of end-of-life service systems in China that will help adapt to the needs of an aging society.

## 2 Methods

### 2.1 Data sources

**Figure 1:**
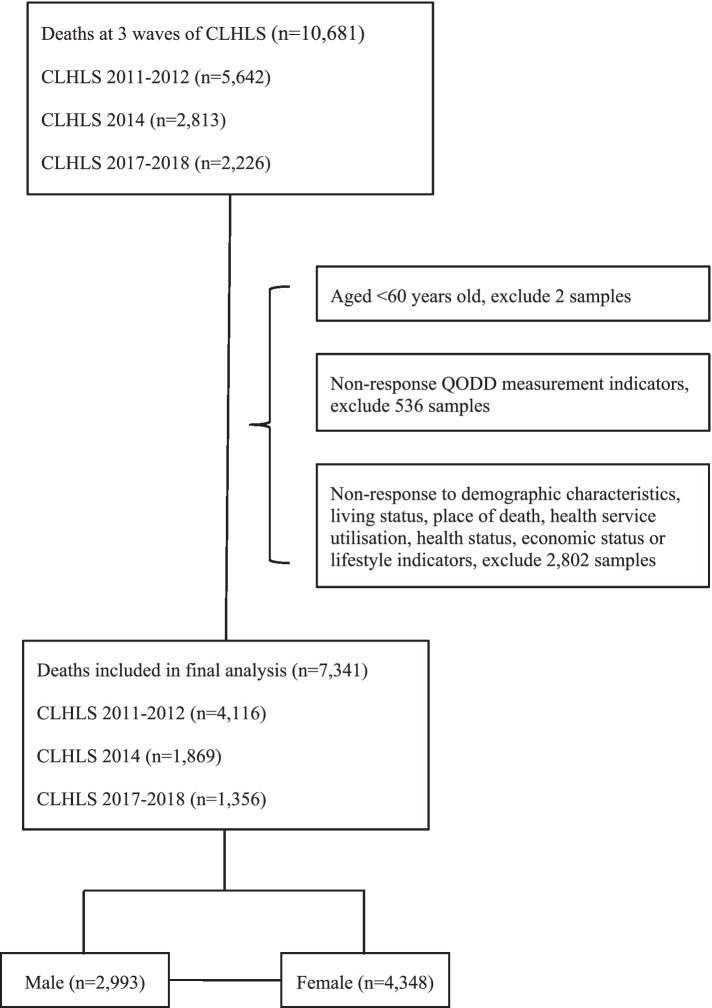
Flowchart of study participants.

This study utilized data from the Chinese Longitudinal Health Longevity Survey (CLHLS), published by the Center for Healthy Aging and Development Studies at Peking University (30). The CLHLS employed a multi-stage stratified sampling method, covering 23 provinces, municipalities, and autonomous regions throughout the country, with a focus on the older adults population aged 80 and above. The survey questionnaires were divided into two types: one for surviving respondents and another for family members of deceased older adults. The baseline survey of CLHLS began in 1998 and was followed by seven waves of tracking surveys in 2000, 2002, 2005, 2008–2009, 2011–2012, 2014, and 2017–2018. When participants passed away between two survey waves, their close contacts (usually family members, occasionally friends, social workers, or others) were interviewed in the next wave to gather information about the deceased’s experiences before and after death. The collected information included medical history before death, cause of death, and circumstances of death. Previous evidence has indicated high data quality of the CLHLS (11). This study selected samples of deceased older adults individuals aged 60 and above from the 2011–2012, 2014, and 2017–2018 waves of CLHLS, merging the three waves of data to construct a cross-sectional dataset of the deceased population from 2008 to 2018. Due to changes in the questionnaire design since the 2011–2012 wave, data from earlier waves could not be included in the analysis. After deleting missing data on important related variables such as QODD, residence, and place of death, the final sample included 7,341 respondents. Of the total, 40.77% were male and 59.23% were female. The data processing procedure is shown in Figure 1. To ensure the representativeness of the data, a comparison of key variables between the original dataset and the final dataset was conducted as a supplementary analysis (see Supplementary Table S1).

### 2.2 QODD measurement

Dupre et al. (31) suggested that the older adults’ ability to pass away peacefully is an important expression of high QODD. Therefore, the degree of pain experienced by the older adults at the time of death is a core indicator for assessing QODD. In this study, we selected the questionnaire “Did the deceased elder feel painful when death was coming?” to measure the QODD of the older adults. The responses to the questionnaire included “very painful,” “relatively painful,” “so so,” “relatively peaceful,” “very peaceful,” and “difficult to say.” Referring to previous studies (32), a value of 1 was assigned to “relatively peaceful” and “very peaceful” to indicate high QODD, while “very painful,” “relatively painful,” “so so,” and “difficult to say” were assigned a value of 0 to indicate low QODD.

### 2.3 Variable description

The study included variables such as demographic characteristics, residential setting, place of death, health service utilization, health status, economic status, and lifestyle, based on previous research on QODD (32–37). Table 1 shows the specific definitions of variables and their assigned values.

**Table 1:** Definition and measurement of variables.

Type	Name	Assignment
Dependent variable	QODD	Low QODD = 0; High QODD = 1
Grouping variable	Sex	Male = 0; Female = 1
Demographic characteristics	Age (years)	60–74 = 1; 75–90 = 2; >90 = 3
	Residence	City = 0; Rural = 1
	Ethnic group	Han = 0; Non-Han
	Marital status	With spouse = 0; ^a^Without spouse = 1
Living status	Living arrangement	Nursing home = 1; Alone = 2; Living with family = 3
	Home facilities score	0–3 = 1; 4–6 = 2; 7 = 3
Place of death	Place of death	Home = 1; Hospital = 2; Institution = 3; Other = 4
Health service utilization	Primary caregiver	^b^Informal caregivers = 1; ^c^Formal caregivers = 2; Nobody = 3
	Sources of medical costs	The government = 1; Family = 2; Medical insurance = 3; No money =4; Was not ill = 5
	Medical costs (RMB)	≤10,000 = 1; 10,001–50,000 = 2; 50,001–100,000 = 3
	Got timely treatment	Yes = 1; No = 2; Was not ill = 3
	Old-age insurance	Yes = 0; No = 1
Health status	Bedridden	No = 0; Yes = 1
	Suffered from serious illness	No = 0; Yes = 1
	Number of chronic diseases	0 = 0; 1 = 1; ≥2 = 2
	^d^ADL score	<12 = 1; ≥12 = 2
	Unconsciousness	No = 0; Yes = 1
	Hearing loss	Yes = 0; No = 1
Economic status	Main financial source	Retirement wage = 1; Family = 2; Local government = 3
	Annual income (RMB)	≤10,000 = 1; 10,001–50,000 = 2; 50,001–100,000 = 3; >100,000 = 4
Lifestyle	Smoking	No = 0; Yes = 1
	Drinking	No = 0; Yes = 1

^aWithout spouse = include never married, divorced and widowed; bInformal caregivers = include relatives and friends; cFormal caregivers = include social service and housekeeper; dADL = activities of daily living.^

### 2.4 Statistical analysis

The statistical analysis was conducted using the SPSS 26.0 software. Descriptive statistical methods were employed to analyze the general data. Additionally, the Chi-square test was performed to analyze the differences in QODD and gender differences in variable distribution among older adults. Forward stepwise regression with logistic regression analysis was used to analyze the QODD variables among Chinese older adults, with gender stratification. The significance level was set at *α* = 0.05.

### 2.5 FDA

The FDA was conducted using StataMP 17.0 to identify sex differences in QODD among older Chinese individuals and their underlying causes. FDA is a frequently employed methodology in studies aimed at ascertaining the impact of disparate factors on a dichotomous dependent variable. The results of previous studies have indicated that FDA is a more effective method for quantifying the contribution and significance of various influencing factors (38, 39). Relevant studies (36, 38) have suggested that the use of FDA in non-linear regression models can effectively quantify the contribution and significance of various factors. The FDA model categorizes the outcomes into two components: explained and unexplained (40, 41). The explained segment of the disparities is attributed to the variables examined in the study, whereas the unexplained segment arises from differences in both measured categorical variables and unobserved factors. Following Fairlie’s approach, the decomposition of the non-linear equation $Y=F\left(X\hat{\beta }\right)$ can be written as follows:


(1) $$\begin{array}{c} {\bar{Y}}^{a}-{\bar{Y}}^{b}=\left[\overset{N^{a}}{\underset{i=1}{\sum }}\frac{F\left(X_{i}^{a}\beta^{b}\right)}{N^{a}}-\overset{N^{b}}{\underset{i=1}{\sum }}\frac{F\left(x_{i}^{b}\beta^{b}\right)}{N^{b}}\right]\\ +\left[\overset{N^{a}}{\underset{i=1}{\sum }}\frac{F\left(x_{i}^{a}\beta^{a}\right)}{N^{a}}-\overset{N^{a}}{\underset{i=1}{\sum }}\frac{F\left(x_{i}^{a}\beta^{b}\right)}{N^{a}}\right] \end{array}$$

The average probabilities of the binary QODD outcomes for the two groups are denoted by ${\bar{Y}}^{a}$ and ${\bar{Y}}^{b}$ , respectively. *F* denotes the cumulative distribution function associated with the logistic distribution. The difference ${\bar{Y}}^{a}-{\bar{Y}}^{b}$ represented the total variation due to group differences. *N*^a^ and *N*^b^ represent the sample sizes of the respective populations. The first term within the parentheses in Equation (1) accounts for the portion of the disparity resulting from differences in observed attributes and variations in estimated coefficients. The second term addresses the contribution to the disparity from differences in Y levels.

## 3 Results

### 3.1 Demographic information of the respondents

Table 2 shows the results of the descriptive statistical analysis contrasting male and female older Chinese individuals. The analysis revealed that 39.38% of older population were in “low” QODD, 60.62% were in “high” QODD, and the proportion of high QODD in females (63.80%) was greater than that in males (56.00%; *p* < 0.001). 79.46% of the female respondents were >90 years old, and 59.64% of the male respondents were >90 years old. 92.71% of female respondents were without a spouse, and 68.26% of male respondents were without a spouse.

**Table 2:** Distribution of the variables in male and female respondents.

Variable	Male [*n*(%)]	Female [*n*(%)]	*χ^2^*	*P*
QODD			45.203	<0.001
Low QODD	1,317 (44.00)	1,574 (36.20)		
High QODD	1,676 (56.00)	2,774 (63.80)		
Age (years)			348.856	<0.001
60–74	128 (4.28)	63 (1.45)		
75–90	1,080 (36.08)	830 (19.09)		
>90	1785 (59.64)	3,455 (79.46)		
Residence			5.273	0.022
City	1,221 (40.80)	1,658 (38.13)		
Rural	1772 (59.20)	2,690 (61.87)		
Ethnic group			1.545	0.214
Han	2,796 (93.42)	4,029 (92.66)		
Non-Han	197 (6.58)	319 (7.34)		
Marital status			742.093	<0.001
With spouse	950 (31.74)	317 (7.29)		
Without spouse	2043 (68.26)	4,031 (92.71)		
Living arrangement			35.969	<0.001
Nursing home	74 (2.47)	109 (2.51)		
Alone	476 (15.90)	483 (11.11)		
Living with family	2,443 (81.62)	3,756 (86.38)		
Home facilities score			2.738	0.254
0–3	598 (19.98)	807 (18.56)		
4–6	1,176 (39.29)	1708 (39.28)		
7	1,219 (40.73)	1833 (42.16)		
Place of death			49.456	<0.001
Home	2,628 (87.80)	4,002 (92.04)		
Hospital	294 (9.82)	245 (5.63)		
Institution	54 (1.80)	88 (2.02)		
Other	17 (0.57)	13 (0.30)		
Primary caregiver			1.653	0.438
Informal caregivers	2,784 (93.02)	4,075 (93.72)		
Formal caregivers	115 (3.84)	156 (3.59)		
Nobody	94 (3.14)	117 (2.69)		
Sources of medical costs			236.774	<0.001
The government	307 (10.26)	122 (2.81)		
Family	1805 (60.31)	3,171 (72.93)		
Medical insurance	831 (27.76)	958 (22.03)		
No money	50 (1.67)	97 (2.23)		
Medical costs (RMB)			160.633	<0.001
≤10,000	2,353 (78.62)	3,879 (89.21)		
10,001–50,000	530 (17.71)	412 (9.48)		
>50,000	110 (3.68)	57 (1.31)		
Got timely treatment			36.853	<0.001
Yes	2,359 (78.82)	3,193 (73.44)		
No	111 (3.71)	141 (3.24)		
Was not ill	523 (17.47)	1,014 (23.32)		
Old-age insurance			60.247	<0.001
Yes	782 (26.13)	806 (18.54)		
No	2,211 (73.87)	3,542 (81.46)		
Bedridden			20.819	<0.001
No	890 (29.74)	1,084 (24.93)		
Yes	2,103 (70.26)	3,264 (75.07)		
Suffered from serious illness			70.130	<0.001
No	1777 (59.37)	2,994 (68.86)		
Yes	1,216 (40.63)	1,354 (31.14)		
Number of chronic diseases			114.361	<0.001
0	983 (32.84)	1938 (44.57)		
1	1,020 (34.08)	1,350 (31.05)		
≥2	990 (33.08)	1,060 (24.38)		
ADL score			22.870	<0.001
<12	1,163 (38.86)	1,453 (33.42)		
≥12	1830 (61.14)	2,895 (66.58)		
Unconsciousness			12.799	<0.001
No	2007 (67.06)	2,739 (62.99)		
Yes	986 (32.94)	1,609 (37.01)		
Hearing loss			0.061	0.804
Yes	1,476 (49.32)	2,157 (49.61)		
No	1,517 (50.68)	2,191 (50.39)		
Main financial source			397.804	<0.001
Retirement wage	606 (20.25)	229 (5.27)		
Family	2,189 (73.14)	3,828 (88.04)		
Local government	198 (6.62)	291 (6.69)		
Annual income (RMB)			5.047	0.168
≤10,000	1,306 (43.64)	2000 (46.00)		
10,001–50,000	1,217 (40.66)	1,694 (38.96)		
50,001–100,000	293 (9.79)	388 (8.92)		
>100,000	177 (5.91)	266 (6.12)		
Smoking			770.295	<0.001
No	2054 (68.60)	4,055 (93.30)		
Yes	939 (31.40)	293 (6.70)		
Drinking			357.716	<0.001
No	2,152 (71.90)	3,875 (89.12)		
Yes	841 (28.10)	473 (10.88)		

The Chi-square test was used to analyze variations among categorical variables. The analysis revealed disparities in the distribution of 17 factors: age, residence, marital status, living arrangement, place of death, sources of medical costs, medical costs, got timely treatment, old-age insurance, bedridden, suffered from serious illness, number of chronic diseases, ADL scores, unconsciousness, main financial source, smoking and drinking. Compared to men, women are older, have a higher proportion of being without a spouse, a higher proportion of dying at home, a higher proportion of medical expenses sourced from the family, a higher proportion of being without illness, and lower proportions of smoking and drinking.

### 3.2 Distribution of QODD variables in female and male respondents

Table 3 illustrates the distribution of variables related to the QODD across male and female participants. The findings indicate that there are significant gender-based disparities in the distribution of several variables. Notably, the variable distribution for hearing loss and annual income significantly impacted QODD only among male participants. Conversely, the distribution of medical costs and drinking significantly influenced QODD exclusively among female participants.

**Table 3:** Distribution of QODD variables under different statuses of QODD.

Variable	Male				Female			
	Low QODD [*n*(%)]	High QODD [*n*(%)]	*χ* ^2^	*P*	Low QODD [*n*(%)]	High QODD [*n*(%)]	*χ* ^2^	*P*
Age (years)			110.973	<0.001			93.570	<0.001
60–74	94 (7.14)	34 (2.03)			33 (2.10)	30 (1.08)		
75–90	564 (42.82)	516 (30.79)			414 (26.30)	416 (15.00)		
>90	659 (50.04)	1,126 (67.18)			1,127 (71.60)	2,328 (83.92)		
Residence			9.105	0.003			9.012	0.003
City	497 (37.74%)	724 (43.20)			554 (35.20)	1,104 (39.80)		
Rural	820 (62.26)	952 (56.80)			1,020 (64.80)	1,670 (60.20)		
Ethnic group			2.518	0.113			0.093	0.760
Han	1,241 (94.23)	1,555 (92.78)			1,456 (92.50)	2,573 (92.75)		
Non-Han	76 (5.77)	121 (7.22)			118 (7.50)	201 (7.25)		
Marital status			20.316	<0.001			38.688	<0.001
With spouse	475 (36.07)	475 (28.34)			166 (10.55)	151 (5.44)		
Without spouse	842 (63.93)	1,201 (71.66)			1,408 (89.45)	2,623 (94.56)		
Living arrangement			17.059	<0.001			22.421	<0.001
Nursing home	34 (2.58)	40 (2.39)			38 (2.41)	71 (2.56)		
Alone	250 (18.98)	226 (13.48)			222 (14.10)	261 (9.41)		
Living with family	1,033 (78.44)	1,410 (84.13)			1,314 (83.48)	2,442 (88.03)		
Home facilities score			36.675	<0.001			55.526	<0.001
0–3	321 (24.37)	277 (16.53)			356 (22.62)	451 (16.26)		
4–6	524 (39.79)	652 (38.90)			664 (42.19)	1,044 (37.64)		
7	472 (35.84)	747 (44.57)			554 (35.20)	1,279 (46.11)		
Place of death			14.324	0.002			15.851	0.001
Home	1,133 (86.03)	1,495 (89.20)			1,428 (90.72)	2,574 (92.79)		
Hospital	144 (10.93)	150 (8.95)			114 (7.24)	131 (4.72)		
Institution	26 (1.97)	28 (1.67)			25 (1.59)	63 (2.27)		
Other	14 (1.06)	3 (0.18)			7 (0.44)	6 (0.22)		
Primary caregiver			1.165	0.558			3.879	0.144
Informal caregivers	1,231 (93.47)	1,553 (92.66)			1,481 (94.09)	2,594 (93.51)		
Formal caregivers	45 (3.42)	70 (4.18)			46 (2.92)	110 (3.97)		
Nobody	41 (3.11)	53 (3.16)			47 (2.99)	70 (2.52)		
Sources of medical costs			2.856	0.414			18.050	<0.001
The government	128 (9.72)	179 (10.68)			38 (2.41)	84 (3.03)		
Family	783 (59.45)	1,022 (60.98)			1,143 (72.62)	2028 (73.11)		
Medical insurance	385 (29.23)	446 (26.61)			375 (23.82)	583 (21.02)		
No money	21 (1.59)	29 (1.73)			18 (1.14)	79 (2.85)		
Medical costs (RMB)			23.282	<0.001			21.462	<0.001
≤10,000	982 (74.56)	1,371 (81.80)			1,359 (86.34)	2,520 (90.84)		
10,001–50,000	280 (21.26)	250 (14.92)			187 (11.89)	225 (8.11)		
>50,000	55 (4.18)	55 (3.28)			28 (1.78)	29 (1.06)		
Got timely treatment			72.693	<0.001			85.386	<0.001
Yes	1,074 (81.55)	1,285 (76.67)			1,226 (77.89)	1967 (70.91)		
No	80 (6.07)	31 (1.85)			84 (5.34)	57 (2.05)		
Was not ill	163 (12.38)	360 (21.48)			264 (16.77)	750 (27.04)		
Old-age insurance			0.244	0.621			0.689	0.406
Yes	350 (26.58)	432 (25.78)			302 (19.19)	504 (18.17)		
No	967 (73.42)	1,244 (74.22)			1,272 (80.81)	2,270 (81.83)		
Bedridden			12.923	<0.001			21.397	<0.001
No	347 (26.35)	543 (32.40)			329 (20.90)	755 (27.22)		
Yes	970 (73.65)	1,133 (67.60)			1,245 (79.10)	2019 (72.78)		
Suffered from serious illness			52.812	<0.001			58.092	<0.001
No	685 (52.01)	1,092 (65.16)			972 (61.75)	2022 (72.89)		
Yes	632 (48.00)	584 (34.85)			602 (38.25)	752 (27.11)		
Number of chronic diseases			77.723	<0.001			54.275	<0.001
0	322 (24.45)	661 (39.44)			601 (38.18)	1,337 (48.20)		
1	487 (36.98)	533 (31.80)			502 (31.89)	848 (30.57)		
≥2	508 (38.57)	482 (28.76)			471 (29.92)	589 (21.23)		
ADL score			15.285	<0.001			22.557	<0.001
<12	460 (34.93)	703 (41.95)			455 (28.91)	998 (35.98)		
≥12	857 (65.07)	973 (58.05)			1,119 (71.09)	1776 (64.02)		
Unconsciousness			38.438	<0.001			40.636	<0.001
No	804 (61.05)	1,203 (71.78)			894 (56.80)	1845 (66.51)		
Yes	513 (38.95)	473 (28.22)			680 (43.20)	929 (33.49)		
Hearing loss			8.889	0.003			0.106	0.745
Yes	609 (46.24)	867 (51.73)			786 (49.94)	1,371 (49.42)		
No	708 (53.76)	809 (48.27)			788 (50.06)	1,403 (50.58)		
Main financial source			3.585	0.167			4.773	0.092
Retirement wage	250 (18.98)	356 (21.24)			98 (6.23)	131 (4.72)		
Family	971 (73.73)	1,218 (72.67)			1,368 (86.91)	2,460 (88.68)		
Local government	96 (7.29)	102 (6.09)			108 (6.86)	183 (6.60)		
Annual income (RMB)			12.312	0.006			7.186	0.066
≤10,000	618 (46.92)	688 (41.05)			764 (48.54)	1,236 (44.56)		
10,001–50,000	516 (39.18)	701 (41.83)			576 (36.59)	1,118 (40.30)		
50,001–100,000	117 (8.88)	176 (10.50)			141 (8.96)	247 (8.90)		
>100,000	66 (5.01)	111 (6.62)			93 (5.911)	173 (6.24)		
Smoking			<0.001	0.988			0.583	0.445
No	904 (68.64)	1,150 (68.62)			1,474 (93.65)	2,581 (93.04)		
Yes	413 (31.36)	526 (31.38)			100 (6.35)	193 (6.96)		
Drinking			0.111	0.739			8.775	0.003
No	951 (72.21)	1,201 (71.66)			1,432 (90.98)	2,443 (88.07)		
Yes	366 (27.79)	475 (28.34)			142 (9.02)	331 (11.93)		

### 3.3 Multivariate analysis of QODD and gender differences

The results of the logistic regression analyses of QODD reported by older Chinese men and women are shown in Tables 4, 5. More detailed results are provided in the Supplementary Tables S2, S3. The results showed that the factors influencing QODD differed between older adults of different genders. Age (75–90, OR = 2.441, 95% CI = 1.597–3.732; >90, OR = 3.779, 95% CI = 2.476–5.768), residence (rural, OR = 0.800, 95% CI = 0.678–0.944), home facilities score (4–6, OR = 1.478, 95% CI = 1.199–1.822; 7, OR = 1.873, 95% CI = 1.507–2.328), place of death (other, OR = 0.165, 95% CI = 0.045–0.606), medical costs (10,001–50,000, OR = 0.753, 95% CI = 0.610–0.930), got timely treatment (no, OR = 0.374, 95% CI = 0.241–0.579; was not ill, OR = 1.380, 95% CI = 1.105–1.724), number of chronic diseases (1, OR = 0.675, 95% CI = 0.555–0.822; ≥2, OR = 0.645, 95% CI = 0.523–0.794), unconsciousness (yes, OR = 0.640, 95% CI = 0.545–0.752) were the factors influencing QODD among male older adults. Meanwhile, residence (rural, OR = 0.868, 95% CI = 0.755–0.999), marital status (without spouse, OR = 1.471, 95% CI = 1.415–1.890), home facilities score (7, OR = 1.707, 95% CI = 1.412–2.062), place of death (hospital, OR = 0.624, 95% CI = 0.472–0.825), got timely treatment (no, OR = 0.480, 95%CI = 0.337–0.684; was not ill, OR = 1.440, 95% CI = 1.215–1.707), bedridden (yes, OR = 0.820, 95% CI = 0.700–0.961), suffered from serious illness (yes, OR = 0.778, 95% CI = 0.671–0.901), unconsciousness (yes, OR = 0.720, 95% CI = 0.629–0.823), drinking (yes, OR = 1.293, 95% CI = 1.044–1.602) were the factors influencing QODD among female older adults. Therefore, the influencing factors of QODD are not consistent between male and female older individuals, mainly manifested in aspects such as age, medical costs, and the number of chronic diseases.

**Table 4:** Logistic regression analysis of QODD reported by male respondents.

Variable	OR	[95% CI]	*P*
Age (years)			
60–74	*Ref*		
75–90	2.441	(1.597,3.732)	<0.001
>90	3.779	(2.476,5.768)	<0.001
Residence			
City	Ref		
Rural	0.800	(0.678,0.944)	0.008
Home facilities score			
0–3	Ref		
4–6	1.478	(1.199,1.822)	<0.001
7	1.873	(1.507,2.328)	<0.001
Place of death			
Home	Ref		
Hospital	0.807	(0.618,1.052)	0.113
Institution	0.764	(0.433,1.349)	0.354
Other	0.165	(0.045,0.606)	0.007
Medical costs (RMB)			
≤10,000	*Ref*		
10,001–50,000	0.753	(0.610,0.930)	0.008
>50,000	1.000	(0.658,1.521)	1.000
Got timely treatment			
Yes	Ref		
No	0.374	(0.241,0.579)	<0.001
Was not ill	1.380	(1.105,1.724)	0.005
Number of chronic diseases			
0	Ref		
1	0.675	(0.555,0.822)	<0.001
≥2	0.645	(0.523,0.794)	<0.001
Unconsciousness			
No	Ref		
Yes	0.640	(0.545,0.752)	<0.001

**Table 5:** Logistic regression analysis of QODD reported by female respondents.

Variable	OR	[95% CI]	*P*
Residence			
City	Ref		
Rural	0.868	(0.755,0.999)	0.049
Marital status			
With spouse	Ref		
Without spouse	1.471	(1.145,1.890)	0.003
Home facilities score			
0–3	Ref		
4–6	1.176	(0.982,1.408)	0.077
7	1.707	(1.412,2.062)	<0.001
Place of death			
Home	Ref		
Hospital	0.624	(0.472,0.825)	0.001
Institution	1.768	(0.865,3.614)	0.118
Other	0.429	(0.138,1.333)	0.143
Got timely treatment			
Yes	Ref		
No	0.480	(0.337,0.684)	<0.001
Was not ill	1.440	(1.215,1.707)	<0.001
Bedridden			
No	Ref		
Yes	0.820	(0.700,0.961)	0.014
Suffered from serious illness			
No	Ref		
Yes	0.778	(0.671,0.901)	0.001
Unconsciousness			
No	Ref		
Yes	0.720	(0.629,0.823)	<0.001
Drinking			
No	Ref		
Yes	1.293	(1.044,1.602)	0.019

### 3.4 FDA results

We performed a quantitative examination to assess the extent to which various factors contribute to the disparities in the QODD reported by male and female individuals in China. The specific results for decomposing the QODD differences are shown in Table 6. The results of the FDA showed that 47.89% of the differences in QODD were caused by the observed variables, while 52.11% of the differences were caused by gender differences and unmeasured variables. Among the variables that accounted for the explained part of the differences, age, number of chronic diseases, suffered from serious illness, unconsciousness, place of death, residence and home facilities scores were influencing factors that reached the level of significance (*p* < 0.05), with contribution levels of 32.95, 12.62, 5.37, −4.13%, 3.66, −2.00 and 0.86%, respectively.

**Table 6:** Fairlie decomposition analysis (FDA) of QODD reported by different genders.

Terms of decomposition	QODD
Difference	−0.0780
Unexplained (%)	−0.0407 (52.11)
Explained (%)	−0.0374 (47.89)

Explained				
Contribution to difference	*P*	*β*	Contribution (%)	[95% CI]
Age (years)	<0.001	−0.0257	32.95	(−0.033, −0.018)
Residence	0.023	0.0016	−2.00	(0.000, 0.003)
Ethnic group	0.359	−0.0002	0.28	(−0.001, 0.000)
Marital status	0.564	−0.0029	3.69	(−0.013, 0.007)
Living arrangement	0.248	−0.0011	1.44	(−0.003, 0.001)
Home facilities score	0.018	−0.0007	0.86	(−0.001, 0.000)
Place of death	0.004	−0.0029	3.66	(−0.005, −0.001)
Primary caregiver	0.291	0.0002	−0.28	(0.000, 0.001)
Sources of medical costs	0.054	0.0007	−0.94	(0.000, 0.001)
Medical costs (RMB)	0.243	−0.0030	3.85	(−0.008, 0.002)
Got timely treatment	0.098	−0.0025	3.23	(−0.006, 0.000)
Old-age insurance	0.710	−0.0006	0.75	(−0.004, 0.003)
Bedridden	0.802	0.0002	−0.30	(−0.002, 0.002)
Suffered from serious illness	0.044	−0.0042	5.37	(−0.008, 0.000)
Number of chronic diseases	<0.001	−0.0098	12.62	(−0.015, −0.005)
ADL score	0.541	0.0006	−0.80	(−0.001, 0.003)
Unconsciousness	<0.001	0.0032	−4.13	(0.002, 0.005)
Hearing loss	0.162	−0.0003	0.41	(−0.001, 0.000)
Main financial source	0.099	0.0045	−5.77	(−0.001, 0.010)
Annual income (RMB)	0.525	0.0002	−0.20	(0.000, 0.001)
Smoking	0.234	0.0057	−7.29	(−0.004, 0.015)
Drinking	0.895	−0.0005	0.59	(−0.007, 0.006)

## 4 Discussion

This investigation aimed to identify the elements affecting the QODD among older Chinese males and females, and it quantitatively assessed the extent of each factor’s influence in order to elucidate the observed gender disparities. The FDA offers clear interpretability and can furnish theoretical insights, aiding pertinent sectors in developing end-of-life care policies tailored for the aging population.

The study results indicated that there were significant gender disparities in QODD among older Chinese individuals, where females exhibiting a higher QODD than men. The results of our study are consistent with those of Neimeyer et al. (33). Studies have shown that older women are generally more health and diet conscious and may be more inclined to make healthy dietary and lifestyle choices (42), which can help to reduce the risk of chronic diseases, thereby improving QODD and reducing the risk of death. In terms of family support, traditional Chinese culture endows women with closer family support networks (43), and they form a multi-generation emotional connection in the process of assuming major family responsibilities. Such social capital can significantly ease anxiety and loneliness in the terminal stage. At the same time, women also show a wider social communication network, and the emotional comfort and companionship from friends constitute an important psychosocial support system (44, 45). In addition, facing the end of life challenges, women tend to adopt positive coping strategies, such as active help seeking and relaxation training, and these adaptive behaviors help to maintain a positive mental state. In contrast, men in the traditional social structure tend to bear more heavy family economic responsibility and social expectations (46), long-term exposure to occupational stress, economic constraints and social competition of multiple stress, the cumulative pressure not only increase the risk of mental health, may also be through the physiological mechanism affect disease prognosis, eventually negative effects on QODD and improve the risk of death.

In the analysis of gender disparities, the determinants of QODD exhibit distinct differences between older men and women. Specifically, age, medical expenses, and the incidence of chronic diseases are factors that uniquely influence the quality of death in older men. In contrast, marital status, the occurrence of serious illnesses, and drinking are significantly associated with the QODD in older women. For older men, the burden of higher medical costs is correlated with a lower QODD, as the financial strain on both the patient and their family can be overwhelming, and insufficient funds may lead to inadequate treatment, thereby worsening the condition (32). Among older women, those without a spouse tend to experience a higher QODD, which may be linked to the traditional role of women in caring for family members and managing domestic responsibilities. Furthermore, alcohol consumption emerges as a protective factor for the QODD in older women, and similar results have been reported internationally (47, 48). Although alcohol is generally recognized as a health risk, this paradox may be explained by the selective nature of health behaviors in the older adults; individuals in poorer health may be more likely to abstain from drinking, while those in better health may continue their established drinking patterns.

The results of FDA demonstrated that age, the number of chronic diseases, suffering from serious illness, unconsciousness, the place of death, residence and home facilities scores were the variables contributing gender disparities in QODD and reached the level of statistical significance. Of these variables, age demonstrated the greatest influence. The results of the multifactorial unconditional logistic regression model analysis indicated that age was a significant factor influencing QODD in older men, with older age being associated with higher QODD. These findings align with Braun (9) that as individuals age, they may become more accepting of mortality and better prepared to confront it. Additionally, the accumulation of life experience and wisdom accompanying aging may enhance an individual’s capacity to derive meaning and value from life (49).

Health conditions, such as the number of chronic illnesses, suffering from serious illness, and unconsciousness, contribute to gender differences in QODD among older adults. In consistency with the findings of previous studies (50, 51), older adults with adverse health conditions may experience physical and psychological pain and discomfort, including pain, dyspnea, insomnia, and anxiety. These symptoms may result in a notable decline in the quality of life of older adults in the final stages of life. Secondly, when older persons suffer from ill health, they may need to be bedridden or dependent on others for care, which puts psychological stress on their families and caregivers and leads to a reduction in social activities and a narrowing of the social circle. This, in turn, can exacerbate feelings of loneliness and loss, thus affecting the QODD. Studies have shown that patients who have received hospice services have a higher QODD than those who have not (52, 53). Therefore, comprehensive care and support for older people in poor health is very important, and in order to improve the QODD of older people, we should provide them with dignified and comfortable end-of-life care.

The findings of our study showed that older adults who died at home had a higher QODD than those who died in other locations. Studies by Yao et al. (54) and Zhao et al. (55) suggest that the QODD of those who died at home was significantly higher than that of patients who died in hospital. Dying at home is typically regarded as the most appropriate and desired location for most individuals (56), as it can evoke a sense of familiarity and comfort, and facilitate a more natural and less medicalized process of dying. Patients who die at home tend to be better equipped to make arrangements and accept their fate, which may contribute to higher QODDs.

Furthermore, the place of residence is also associated with the QODD. The results of this study demonstrate that the QODD is higher among the older adults who reside in urban areas, which is consistent with the findings of previous studies (13, 43). This may be due to the fact that urban areas have richer health care resources and facilities. Studies have shown that the higher the economic level, the better the health care, the easier it is to meet the medical and nursing needs of the older adults before they die (57, 58), and therefore the higher the QODD of the older adults. Additionally, residents in urban areas may be more likely to have access to amenity services, which may reduce the burden of living on patients and their families and help improve QODD. These findings served as a reference for relevant departments to reduce gender differences in QODD among Chinese older adults from a more holistic perspective.

Based on the above research findings, we make the following recommendations. Firstly, it is imperative to prioritize the health status of the older population. There is a need for widespread promotion of chronic disease self-management education tailored for the older adults, aimed at enhancing their disease awareness and self-care capabilities, particularly for conditions more prevalent among older males. The government should strengthen collaborations with healthcare institutions to facilitate accessible and affordable health screening services for the older adults facilitating early detection and treatment of diseases. For those older individuals with severe illnesses, appropriate adjustments in health insurance policies should be made, and targeted assistance and support programs should be developed. Additionally, a specialized service team, comprising medical professionals, nurses, social workers, and volunteers, should be established to offer comprehensive support to older individuals with cognitive impairments. Secondly, there is an urgency to develop community-based end-of-life care services, providing professional care to the older adults and encouraging them to articulate their preferences and wishes. Thirdly, the government should increase its support for rural areas, promoting the adaptation of household facilities for the older adults to enhance the safety and comfort of aging in place, thereby narrowing the urban–rural gap. Finally, the healthcare system should broadly implement gender sensitivity education to augment healthcare providers’ understanding of gender differences, and to offer professional training to family members to improve their knowledge and skills in this domain.

Our study also has a few limitations. First, the QODD has several dimensions. “Did the deceased elder feel painful when death was coming?” is a subjective measure that is influenced by the respondents. As a result, it has a limited reflection of the objective QODD. Second, the QODD of the older adults is influenced by several factors, and we have only measured some of them. In the future, we will use more objective measurement tools, include more factors in our analyses, and conduct studies in larger populations to validate the validity of our findings. Third, the questionnaire was filled out by the relatives of the deceased older adults, and we did not measure the relevant information of the respondents, which may have some bias. Future studies should examine whether there are differences in the assessment of death quality among different relatives. Despite these limitations, the results are useful for comparing the differences in the QODD between the older adults of different genders in China, and providing guidelines for improving the QODD among the older adults in China.

## 5 Conclusion

This study analyzed the differences between men and women in terms of QODD in China. We found that Chinese men have a poorer QODD compared to women. The main factors contributing to this difference were age, the number of chronic diseases, suffered from serious illness, unconsciousness, place of death, residence and home facilities scores. Therefore, the relevant departments should focus on addressing these factors to reduce the gap between men and women. Our findings help in comprehensively improving the QODD in older adults individuals and provide guidance for the implementation of the Healthy China Strategy to promote successful aging.

## Data Availability

The datasets presented in this study can be found in online repositories. The names of the repository/repositories and accession number(s) can be found: https://opendata.pku.edu.cn/dataset.xhtml?persistentId=doi:10.18170/DVN/WBO7LK.
