# Supplementary material for: Gender differences in quality of dying and death among older adults: a cross-sectional study in China
Source: Front Public Health. 2025 Mar 5;13:1542918. doi: 10.3389/fpubh.2025.1542918 (PMC11919661; doi:10.3389/fpubh.2025.1542918)
Supplement: Supplementary file 1 [file Data_Sheet_1.pdf]

Supplementary Material

1 Supplementary Tables

Table S1 Comparison of key variables between the original and final datasets

Variable	Original dataset (n=10,681)	Final dataset (n=7,341)	*t/χ2*	*P*
Age(years)	94.51±9.007	94.60±8.842	-0.655	0.512
Sex				0.883
Male	4,343(40.66)	2,993(40.77)	0.022	
Female	6,338(59.34)	4,348(59.23)		
Residence			0.153	0.696
City	4,158(38.93)	2,879(39.22)		
Rural	6,523(61.07)	4,462(60.78)	0.153	
Ethnic group			0.471	0.492
Han	9,588(89.77)	6,825(92.97)		
Non-Han	755(7.07)	516(7.03)		
Marital status			1.077	0.584
With spouse	1,862(17.43)	1,267(17.26)		
Without spouse	8,708(81.53)	6,074(82.74)		

Table S2 Logistic regression analysis of QODD reported by male respondents

Variable	b	*P*	OR	[95% CI]
Age (years)				
60-74			*Ref*	
75-90	0.892	<0.001	2.441	(1.597,3.732)
>90	1.330	<0.001	3.779	(2.476,5.768)
Residence				
City			*Ref*	
Rural	-0.223	0.008	0.800	(0.678,0.944)
Home facilities score				
0-3			*Ref*	
4-6	0.391	<0.001	1.478	(1.199,1.822)

7	0.628	<0.001	1.873	(1.507,2.328)
Place of death				
Home			*Ref*	
Hospital	-0.215	0.113	0.807	(0.618,1.052)
Institution	-0.269	0.354	0.764	(0.433,1.349)
Other	-1.800	0.007	0.165	(0.045,0.606)
Medical costs(RMB)				
≤10,000			*Ref*	
10,001-50,000	-0.284	0.008	0.753	(0.610,0.930)
>50,000	0.000	1.000	1.000	(0.658,1.521)
Got timely treatment				
Yes			*Ref*	
No	-0.984	<0.001	0.374	(0.241,0.579)
Was not ill	0.322	0.005	1.380	(1.105,1.724)
Number of chronic diseases				
0			*Ref*	
1	-0.392	<0.001	0.675	(0.555,0.822)
≥2	-0.439	<0.001	0.645	(0.523,0.794)
Unconsciousness				
No			*Ref*	
Yes	-0.446	<0.001	0.640	(0.545,0.752)
Constant	-0.645	0.010	0.524	

Table S3 Logistic regression analysis of QODD reported by female respondents

Variable	b	*P*	OR	[95% CI]
Age (years)				
60-74			*Ref*	
75-90	-0.036	0.895	0.965	(0.566,1.644)
>90	0.489	0.069	1.630	(0.962,2.763)
Residence				
City			*Ref*	
Rural	-0.141	0.049	0.868	(0.755,0.999)
Marital status				
With spouse			*Ref*	

Without spouse	0.386	0.003	1.471	(1.145,1.890)
Living arrangement				
Nursing home			*Ref*	
Alone	0.120	0.714	1.128	(0.593,2.146)
Living with family	0.370	0.242	1.448	(0.778,2.693)
Home facilities score				
0-3			*Ref*	
4-6	0.162	0.077	1.176	(0.982,1.408)
7	0.535	<0.001	1.707	(1.412,2.062)
Place of death				
Home			*Ref*	
Hospital	-0.471	0.001	0.624	(0.472,0.825)
Institution	0.570	0.118	1.768	(0.865,3.614)
Other	-0.847	0.143	0.429	(0.138,1.333)
Got timely treatment				
Yes			*Ref*	
No	-0.734	<0.001	0.480	(0.337,0.684)
Was not ill	0.365	<0.001	1.440	(1.215,1.707)
Bedridden				
No			*Ref*	
Yes	-0.199	0.014	0.820	(0.700,0.961)
Suffered from serious illness				
No			*Ref*	
Yes	-0.251	0.001	0.778	(0.671,0.901)
Unconsciousness				
No			*Ref*	
Yes	-0.329	<0.001	0.720	(0.629,0.823)
Drinking				
No			*Ref*	
Yes	0.257	0.019	1.293	(1.044,1.602)
Constant	-0.393	0.359	0.675	
